# An integrated package for bisulfite DNA methylation data analysis with Indel-sensitive mapping

**DOI:** 10.1186/s12859-018-2593-4

**Published:** 2019-01-22

**Authors:** Qiangwei Zhou, Jing-Quan Lim, Wing-Kin Sung, Guoliang Li

**Affiliations:** 1 National Key Laboratory of Crop Genetic Improvement, Agricultural Bioinformatics Key Laboratory of Hubei Province, College of Informatics, Huazhong Agricultural University, Wuhan, 430070 China; 2 Department of Computer Science, National University of Singapore, Singapore, 117417 Singapore; 3 Department of Computational and Systems Biology, Genome Institute of Singapore, Singapore, 138672 Singapore; 4 Agricultural Bioinformatics Key Laboratory of Hubei Province, College of Informatics, Huazhong Agricultural University, Wuhan, 430070 China; 5 Lymphoma Genomic Translational Research Laboratory, National Cancer Centre, Singapore, Singapore

**Keywords:** DNA methylation, Bisulfite sequencing, Alignment, Indel, Pipeline

## Abstract

**Background:**

DNA methylation plays crucial roles in most eukaryotic organisms. Bisulfite sequencing (BS-Seq) is a sequencing approach that provides quantitative cytosine methylation levels in genome-wide scope and single-base resolution. However, genomic variations such as insertions and deletions (indels) affect methylation calling, and the alignment of reads near/across indels becomes inaccurate in the presence of polymorphisms. Hence, the simultaneous detection of DNA methylation and indels is important for exploring the mechanisms of functional regulation in organisms.

**Results:**

These problems motivated us to develop the algorithm BatMeth2, which can align BS reads with high accuracy while allowing for variable-length indels with respect to the reference genome. The results from simulated and real bisulfite DNA methylation data demonstrated that our proposed method increases alignment accuracy. Additionally, BatMeth2 can calculate the methylation levels of individual loci, genomic regions or functional regions such as genes/transposable elements. Additional programs were also developed to provide methylation data annotation, visualization, and differentially methylated cytosine/region (DMC/DMR) detection. The whole package provides new tools and will benefit bisulfite data analysis.

**Conclusion:**

BatMeth2 improves DNA methylation calling, particularly for regions close to indels. It is an autorun package and easy to use. In addition, a DNA methylation visualization program and a differential analysis program are provided in BatMeth2. We believe that BatMeth2 will facilitate the study of the mechanisms of DNA methylation in development and disease. BatMeth2 is an open source software program and is available on GitHub (https://github.com/GuoliangLi-HZAU/BatMeth2/).

**Electronic supplementary material:**

The online version of this article (10.1186/s12859-018-2593-4) contains supplementary material, which is available to authorized users.

## Background

DNA methylation is an important epigenetic modification that plays critical roles in cellular differentiation [1], genomic imprinting [2], X-chromosome inactivation [3], development [4] and disease [5]. Bisulfite sequencing applies a bisulfite treatment to genomic DNA to convert nonmethylated cytosines to uracils, which can be sequenced as thymines (T). Methylated cytosines cannot be converted to uracils and are sequenced as cytosines (C). In this way, methylated and nonmethylated Cs can be distinguished. Whole-genome bisulfite sequencing (BS-Seq) is a method to convert nonmethylated cytosines into thymines for DNA methylation detection at single-base resolution, a process that has substantially improved DNA methylation studies. However, bisulfite conversion introduces mismatches between the reads and the reference genome, which leads to slow and inaccurate mapping. In the last few years, a number of tools have been developed for BS-read alignment, such as BatMeth [6], BSMAP [7], Bismark [8], BS-Seeker2 [9], BWA-meth [10], BSmooth [11] and Biscuit [12].

Structural variations (SVs) play a crucial role in genetic diversity [13–15]. Many SVs are associated with cancers and genetic diseases such as psoriasis, sporadic prostate cancer, high-grade serous ovarian cancer and small-cell lung cancer [16–18]. Insertions and deletions (indels) are the second most common type of human genetic variants after single nucleotide polymorphisms (SNP) [19]. Many human inherited diseases have been reported to be related to indels [20, 21]. Recent results show that the indel rate in the human genome is approximately 1 in 3000 bp [22]. If we cannot align indel-containing reads accurately, the resulting misalignments can lead to numerous errors in the downstream data analysis and directly affect the calling of DNA methylation, which leads to incorrect results. Because DNA methylation and indels both play important roles in development and diseases such as cancer, it is necessary to detect them simultaneously.

However, the current methylation callers fail to accurately align reads to indel regions. BSMAP can detect only indels with lengths less than 3 nucleotides. Other tools, such as BWA-meth (which uses BWA-mem [23] as the fundamental mapping tool), use seeding approaches. These methods assume that the seeds have no indels. Hence, they cannot obtain the correct results when sequencing reads contain multiple mismatches and indels. As a result, we were motivated to study the alignment performance of the published methods on reads with and without indels. Based on the ‘Reverse-alignment’ and ‘Deep-scan’ ideas in BatAlign [24], we developed the DNA methylation mapping tool BatMeth2, which is sensitive to indels in bisulfite DNA methylation reads. In addition, we also provided programs for DNA methylation data annotation, visualization and differentially methylated cytosine/region (DMC/DMR) detection to facilitate DNA methylation data analysis. The package BatMeth2 is designed to be an easy-to-use, autorun package for DNA methylation analyses.

## Implementation

### Bisulfite sequencing read alignment with BatMeth2

The basic alignment tool underlying BatMeth2 is the alignment program BatAlign [24], which works as follows. First, converted reference genomes and converted input sequences are prepared with all Cs in the reference genomes, and input sequences are converted to Ts. Because the plus and minus strands are not complementary after Cs are converted to Ts, two converted reference genomes are prepared, where one is for the plus strand of the original reference genome and the other is for the minus strand of the original reference genome. The indexes are built for these two converted reference genomes. Many existing approaches first find putative hits for the short seeds of the input reads by performing exact alignment or 1-mismatch alignment of the seeds. When the short seeds have two or more mutations, the putative hits of the short seeds may not represent the correct locations of the input reads. To address the limitation of missing alignment hits with low edit-distance short seeds, BatMeth2 finds hits of long seeds from the input reads allowing a high edit-distance (long seeds of 75 bp, five mismatches and one gap allowed). When the input sequence is shorter than 150 bp, the candidate hits of the 75 bp seed are searched and then extended to their original full read length. When the input read is longer than 150 bp, multiple nonoverlapping 75 bp seeds are used to search for candidate hits. These hits are extended, and then, the best alignment is selected on the basis of a set of predefined criteria, including the mismatch number and the number of mapping hits. For the calculation of the alignment score, the penalty for a gap is exactly the same as the penalty for 1.5 mismatches. If the number of “detected mismatches” in a read is smaller than the mismatch threshold, the detection of indels will not be conducted unless there is no appropriate alignment result for the read. (When there is a mismatch alignment of a read with a small number of mismatches, it is better than an alignment with indels. Hence, it is unnecessary to obtain a gapped alignment.) When the allowed number of mismatches is greater than the mismatch threshold, BatMeth2 will detect indels and report the alignment hit. This algorithm will not sacrifice accuracy, yet it is more efficient. Additional file 1: Figure S1 outlines the details of the BatMeth2 algorithm.

The final alignment between a read and the reference genome is based on an affine-gap scoring scheme, where the score for a match or a mismatch is the Phred scaled value at this position. The gap opening penalty and the gap extension penalty are 40 and 6, respectively.

In reduced representation bisulfite sequencing (RRBS), the genomic DNA is first fragmented by enzymatic digestion (e.g., MspI), followed by a size selection step to enrich the fragments for CpG islands. Therefore, in BatMeth2, we partition the genome by enzymatic digestion site (e.g., C-CGG for MspI); then, we index only the reduced representation genome regions, which are fragment regions that are shorter than the predefined value, which is 600 by default. We map the RRBS reads by building special enzymatic digestion indexes with improved efficiency.

### Methods for aligning reads across the breakpoints of small insertions and deletions (indels)

BatMeth2 starts scanning for the most likely hits for a read in the reference genome by using ‘Reverse-alignment’. The current alignment methods mostly use seed-and-extend approaches. They first align short seeds allowing 0 or 1 mismatch; then, the seeds are extended. When the alignment of the read contains multiple mismatches and/or indels, the current solutions may fail. To avoid this problem, our approach is to align a long seed (default 75 bp) allowing more mismatches and gaps (by default, we allowed five mismatches and one gap). In addition, for aligning paired-end reads, the best hit for an individual read is not necessarily the best alignment result for the paired-end reads. In this case, we need to consider the alignment results of both reads at the same time. Therefore, after we obtain the least-cost (highest Smith-Waterman score) hit for each read, we continue to search for more alignment hits and finally choose the appropriate alignment results according to the paired-end sequence alignment. This method is called ‘Deep-scan’ and is described in BatAlign [24]. Among the hits of both reads, BatMeth2 finds the best hit pair and reports it.

If a read spans a genomic rearrangement breakpoint, many mismatches between the read and the genome may occur, which will cause the alignment score to be negative. In this case, we will remove some part of this read (soft-clipping). When the soft-clipped length is greater than 20, we will realign the clipped portion of the read (allowing for 0 mismatches) and obtain auxiliary alignments. The chosen auxiliary alignment and the primary alignment of the read together will represent a complete alignment of the original read.

The main differences between BatMeth2 and BatMeth are as follows: 1) BatMeth2 supports gapped alignment with an affine-gap scoring scheme, while BatMeth finds only ungapped alignments. 2) BatMeth2 supports paired-end alignment, while BatMeth can align only single-end reads. 3) BatMeth2 supports characterizing the alignment hits with a mapping quality report. 4) BatMeth2 supports local alignment, which does not require reads to align end-to-end. Therefore, BatMeth2 can remove some part of this read (soft-clipping) based on the alignment score.

### Calculation of methylation levels

To calculate the methylation density, we first count the total number of C/T nucleotides that overlap with each cytosine site on the plus strand and the number of G/A nucleotides on the minus strand. Those cytosines, which are used for further statistical analysis, should meet the criterion that their depth (C plus T) should be more than some predefined threshold (by default, 5) to reduce the influence of sequencing errors in the cytosine site. In addition, we know that there may be a SNP variation from cytosine (C) to thymine (T), which may affect the calculation of methylation levels in the cytosine loci. To determine whether a site contains a C-to-T bisulfite conversion or a C-to-T SNP, we need to consider the reverse complement strand simultaneously. If the cytosine site is a methylation, it will change from C to T after bisulfite treatment, while the reverse complement strand (rev) should be G. Conversely, if the site is a C-to-T SNP, the rev should be A. Therefore, we calculate the methylation level (ML) by the following equation, which was used in the BSMAP [7] program:

$$ \mathrm{ML}=\mathit{\min}\left(\frac{C}{\left(C+T\right)\ast \frac{\mathit{\operatorname{Re}}{v}_G}{\left(\mathit{\operatorname{Re}}{v}_G+\mathit{\operatorname{Re}}{v}_A\right)}},1.0\right)\ast 100\% $$

where C (or T) is the coverage of C (or T) from the reads on the plus strand and RevG (or RevA) is the coverage of G (or A) from the reads on the minus strand.

However, to ensure the accuracy of the DNA ML, the above formula is applied when the coverage on the complement strand of the cytosine site is high. When the coverage on the reverse complementary strand (G + A) is smaller than the preset coverage threshold (default: 10), we calculate the ML by the following equation:

$$ \mathrm{ML}=\frac{C}{\left(C+T\right)}\ast 100\% $$

### Identification of differentially methylated regions (DMRs)

BatMeth2 integrates several commonly used methods for detecting differentially methylated regions (DMRs), for example, the beta-binomial distribution model [25] for data with replicates and Fisher’s exact test for data without replicates. In addition, BatMeth2 can not only scan the whole genome for DMRs but also operate on predefined windows, such as gene bodies, transposable elements (TEs), untranslated regions (UTRs), and CpG islands.

For each sliding window or predefined window, differential analysis can be performed if it meets the following criteria: (1) the region contains at least m valid CpG (or non-CpG) sites (e.g., m = 5) in both samples; (2) each valid CpG site is covered by at least n bisulfite sequencing reads (e.g., *n* = 5). Users can choose a suitable statistical method to perform hypothesis tests. Each predefined window or sliding window acquires one *p* value from the selected statistical testing method. Finally, the *p* values are adjusted with the false discovery rate (FDR) method for multiple hypothesis testing, proposed by Benjamini and Hochberg [26]. If the adjusted *p* value of a window is less than the predefined threshold, and the difference of DNA ML between the two samples is greater than the preset threshold, the window is defined as a DMR.

### Visualization of DNA methylation data

To visualize the methylation profile, the ML in each genomic region is calculated. These genomic regions can be gene bodies or promoters, etc.

To calculate the methylation density level in a given genomic region, only cytosines with coverage greater than the preset threshold are used. The ML in a genomic region is defined as the total number of sequenced Cs over the total number of sequenced Cs and Ts at all cytosine positions across the region, and the equation is as follows:

$$ \mathrm{M}=\frac{\sum_1^nC}{\sum_1^n\left(C+T\right)}\ast 100\% $$

where *n* is the total number of cytosine sites whose coverage is more than the predefined threshold in the genomic region.

### Mapping programs and environment for evaluation

We evaluated the performance of BatMeth2 by aligning both simulated and real BS reads to the human genome (hg19) and compared it with the current popular DNA methylation mapping tools, such as Bismark (v0.14.5), BSMAP (v2.74), BS-Seeker2 (v2.0.8), BWA-meth, BSmooth (v0.8.1) and Biscuit (v0.3.8). All tests were conducted in a workstation with an Intel(R) Xeon(R) E5–2630 0 @ 2.30 GHz CPU and 128 GB RAM running Linux (Red Hat 4.4.7–11). We allowed the same number of mismatches for the read alignment and the same number of CPU threads for all the compared programs in our experiments. If not specified, the parameters were kept as default. When running Bismark (with Bowtie2 as the fundamental mapping method), we used the default parameters and set the alignment seed length as 15 for testing. The format of the BSmooth alignment results was adjusted using the code of BWA-meth.

## Result

### An easy-to-use, autorun package for DNA methylation analyses

**Fig. 1:**
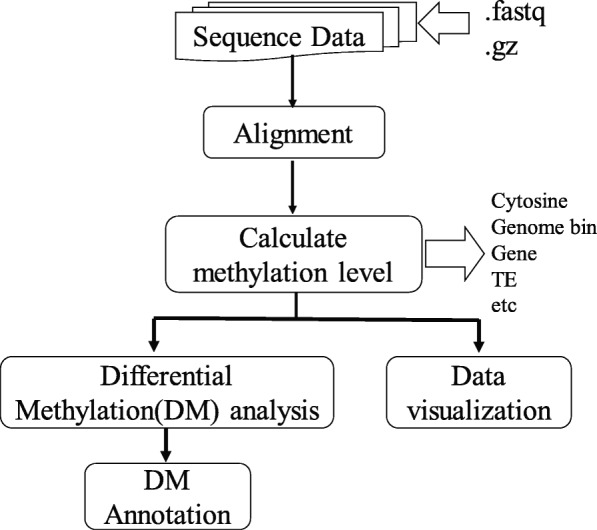
The workflow of BatMeth2. The two big arrows mean input or output files

To complete DNA methylation data analysis more conveniently, we packaged all the functions in an easy-to-use, autorun package for DNA methylation analysis. Figure 1 shows the main features of BatMeth2: 1) BatMeth2 has efficient and accurate alignment performance. 2) BatMeth2 can calculate the DNA methylation level (ML) of individual cytosine sites or any functional regions, such as whole chromosomes, gene regions, transposable elements (TEs), etc. 3) After the integration of different statistical algorithms, BatMeth2 can perform differential DNA methylation analysis for any region, any number of input samples and user requirements. 4) By integrating BS-Seq data visualization (DNA methylation distribution on chromosomes and genes) and differential methylation annotation, BatMeth2 can visualize the DNA methylation data more clearly. During the execution of the BatMeth2 tool, an html report is generated for the statistics of the sample. Sample html report details are shown in http://htmlpreview.github.io/?https://github.com/GuoliangLi-HZAU/BatMeth2/blob/master/BatMeth2-Report/batmeth2.html.

### BatMeth2 has better mapping performance on simulated BS-Seq data

We first evaluated all the aligners using simulated datasets (without indels) consisting of reads with 75 base pairs (bp), 100 bp and 150 bp and with different bisulfite conversion rates (ranging from 0 to 100% with step 10%). These datasets were simulated from the human genome (UCSC hg19) using FASTX-mutate-tools [27], wgsim (v0.3.0) and the simulator in SAMtools (v1.1) [28], which allows 0.03% indels, a 1% base error rate in the whole genome and a maximum of two mismatches per read. We mapped the simulated reads to the reference genome, allowing at most two mismatches. Because the original positions of the simulated reads were known, we could evaluate the accuracy of all the programs by comparing their mapping outputs with the original positions.

**Fig. 2:**
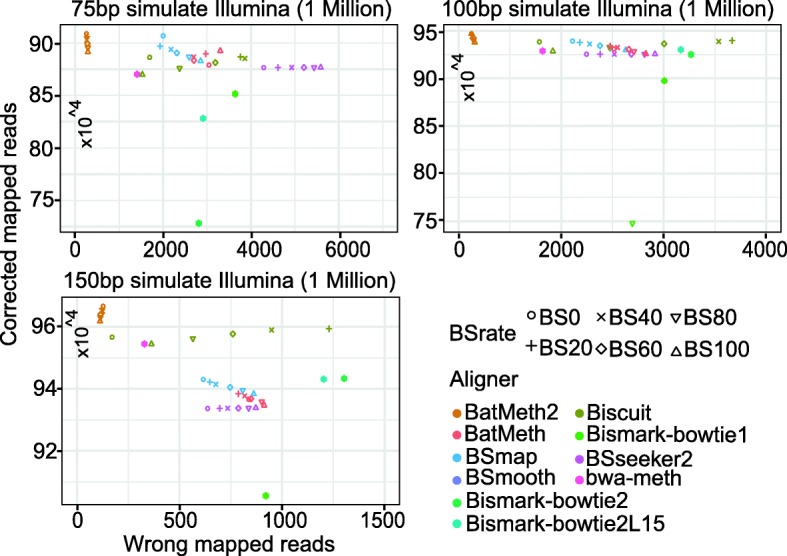
Evaluation of all BS-Seq aligners using simulated datasets with different read lengths from FASTX and wgsim. Simulated data with different bisulfite conversion rates is shown in different shapes. Results from different aligners are shown with different colors of the symbols. The results near the top-left corner in each panel show that the software achieved more number of correctly mapped reads and the lower number of incorrectly mapped reads. The results from our aligner BatMeth2 are the best in the different simulated bisulfite datasets

To compare the performances of the different software, a sequencing read with indels was considered correctly mapped if the following conditions were true: 1) the read was uniquely mapped to the same strand as it was simulated from and the mapping quality was greater than 0; 2) the reported starting position of the aligned read was within ten base pairs of the original starting position of the simulated read; 3) the mapping results had similar indels or mismatches to the simulated read. If any of these conditions were violated, the read was considered wrongly mapped. Because BatMeth2 allows one gap in the seed region, it can find seed locations incorporating indels with high accuracy and can avoid mismatched locations, which would cause reads incorporating indels to be misaligned. The results in Fig. 2 show that BatMeth2 achieved the largest number of correctly aligned reads and the lowest number of incorrectly aligned reads in all test datasets at different bisulfite conversion rates.

In brief, the results from wgsim-simulated indel-aberrant datasets show that BatMeth2 has better performance (1~2% better than the second top aligner) than the other methods when aligning general simulated BS reads containing a mixture of mismatches and indels. We can see that with the increased BS conversion rate, the alignment accuracy of all the software is reduced. In these different conditions, BatMeth2 performs better.

### BatMeth2 has better mapping performance on real BS-Seq data

**Fig. 3:**
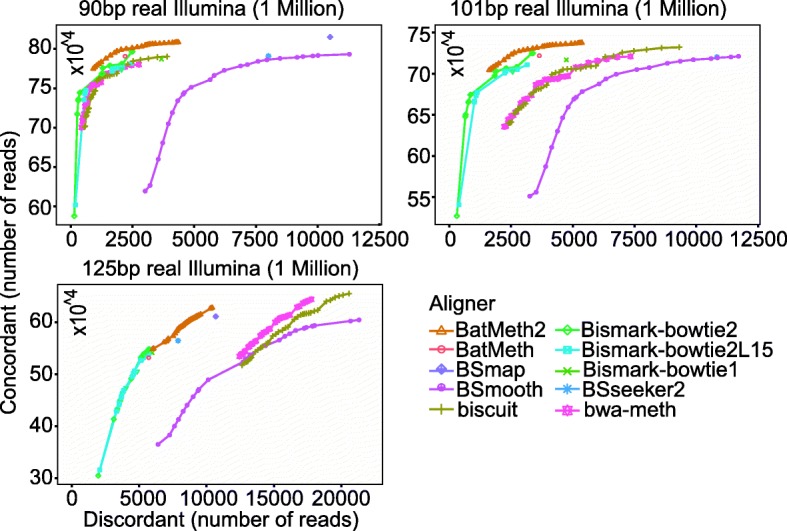
Concordance and discordance rates of alignments on real paired-end reads from different aligners. Cumulative counts of concordant and discordant alignments from high to low mapping quality for real bisulfite sequencing reads. There is only one point for BSmap and the aligners based bowtie separately, since these aligners have no map quality score. Bismark-bowtie2L15 means bowtie2 alignment with seed length 15

To test the performance of BatMeth2 on real BS-Seq datasets, we downloaded paired-end BS-Seq datasets and randomly extracted 1 million 2 × 90 bp paired-end reads from SRA SRR847318, 1 million 2 × 101 bp paired-end reads from SRA SRR1035722 and 1 million 2 × 125 bp paired-end reads from SRA SRR3503136 for evaluation purposes. Because these datasets are from healthy cell lines or tissues, they are expected to contain a low number of structural variations. Hence, we aligned these real data using single-end reads from the paired-end datasets and evaluated the concordant and discordant mapping rates from the paired alignments to estimate the correct and incorrect alignment rates. Because the insert size of the paired-end reads was approximately 500 bp, a pair of partner reads could be considered concordant if they were mapped within a nominal distance of 500 bp; otherwise, a pair of partner reads could be considered discordant. Similar to our results with the simulated data, BatMeth2 reported more concordant and fewer discordant alignments on the real datasets over a large range of map quality scores, as shown in Fig. 3.

In addition, Table 1 shows the relative runtimes of the programs. BatMeth2 with the default settings ran faster than most of the published aligners and was comparable to BWA-meth and BatMeth. Bismark2 (with Bowtie2 as the fundamental mapping method), BS Seeker2 and BSmooth require longer running times.

**Table 1:** Running time (in seconds) from different aligners for real bisulfite reads with length 90 bp

BatMeth	BatMeth2	Bismark-b1	Bismark-b2	Bismark-L15
456	681	633	1869	2102
BWA-Meth	Biscuit	BS Seeker	BSmap	BSmooth
498	1256	1173	774	3740

### DNA methylation calling

To evaluate the accuracy of DNA methylation calling among different software, we downloaded 450 K bead chip data from the IMR90 cell line from ENCODE (Encyclopedia of DNA Elements). We also downloaded whole-genome bisulfite sequencing (WGBS-Seq) data of the IMR90 cell line from ENCODE (42.6 Gbases). For each software, we aligned the WGBS-Seq reads and calculated the level of DNA methylation. Then, we compared the results with the MLs at the same sites in the 450 K Bead Chip data. When the difference between the DNA ML from the WGBS-Seq data by the software and that from the 450 K Bead Chip was less than 0.2, the calling result was defined as correct; otherwise, it was considered incorrect.

The results are shown in Table 2. The overlap among the correct results of all the software is shown in Additional file 1: Figure S2. We can see that BatMeth2 and Biscuit have similar performances, which are better than those of the other software. In conclusion, BatMeth2 improves the accuracy of both BS-read alignment and DNA ML calling.

**Table 2:** Results of methylation calling

450 K (48421)	BatMeth2	Biscuit	bwameth	BSmap	Bismark-b2	Bismark-L15	Bismark-b1
Detected	379,139	379,209	378,256	374,735	364,581	364,518	352,995
	78.59%	78.60%	78.40%	77.68%	75.57%	75.56%	73.17%
Correct	320,650	320,549	319,634	316,620	307,121	307,058	297,399
	66.47%	66.45%	66.26%	65.63%	63.66%	63.65%	61.65%
Wrong	58,489	58,660	58,622	58,115	57,460	57,460	55,596
	12.12%	12.16%	12.15%	12.05%	12.91%	11.91%	11.52%

### BatMeth2 aligns BS reads while allowing for variable-length indels

Cancer contains a notably higher proportion of indels than healthy cells do. Therefore, to verify whether BatMeth2 can align BS reads with indels of different lengths, we downloaded WGBS data (75 Gbases) and 450 K Bead Chip data from HepG2 (liver hepatocellular carcinoma, a cancer cell line) from ENCODE. We checked the indel length distribution in the reads after the alignment of HepG2 WGBS-Seq data. Additional file 1: Figure S3A shows that the lengths of the detected indels were mainly distributed in the 1 bp~ 5 bp range, and the longest indel was 40 bp in length. According to our statistics, 2.3% of the alignment reads contained indels. From these results, we know that BatMeth2 can align reads with indels of different lengths.

Next, we tested the effect of indel detection on DNA methylation calling. For BatMeth2, we ran two options on the HepG2 data: with and without indel detection (i.e., set -I parameter in BatMeth2). We also ran Bismark on the WGBS-Seq data from HepG2 as a reference for DNA methylation calling with indel detection, because Bismark does not have an indel calling function. We compared the calling of DNA methylation in BatMeth2 and Bismark with the calling from the 450 K Bead Chip data. The results are shown in Additional file 1: Figure S3B, where “BatMeth2-noIndel” corresponds to BatMeth2 with no indel detection. We can see that, in the absence of indel detection, the result of BatMeth2 was only slightly better than that of Bismark (with Bowtie1 as the fundamental mapping method). The result of BatMeth2 with indel detection was significantly better. Furthermore, we can see that BatMeth2 can detect more DNA methylation sites than BatMeth2-noIndel and Bismark (Bowtie 1). To understand why the performance of BatMeth2 with indel detection is better, we defined the methylation sites called by BatMeth2 as Result A, while the methylation sites called by BatMeth2-noIndel and Bismark were defined as Result B. Then, we let mclA be the methylation sites appearing in Result A but not Result B. We observed that mclA included 23,853 DNA methylation sites and 15,048 (63%) of the 23,853 sites covered by the alignments of indel reads called by BatMeth2 with indel detection (see Additional file 1: Figure S3C). In addition, we found that the indel rates in Result A and Result B were only 5 and 0%, respectively. Hence, we concluded that accurate indel detection can improve DNA methylation calling.

### Visualization of DNA methylation data

**Fig. 4:**
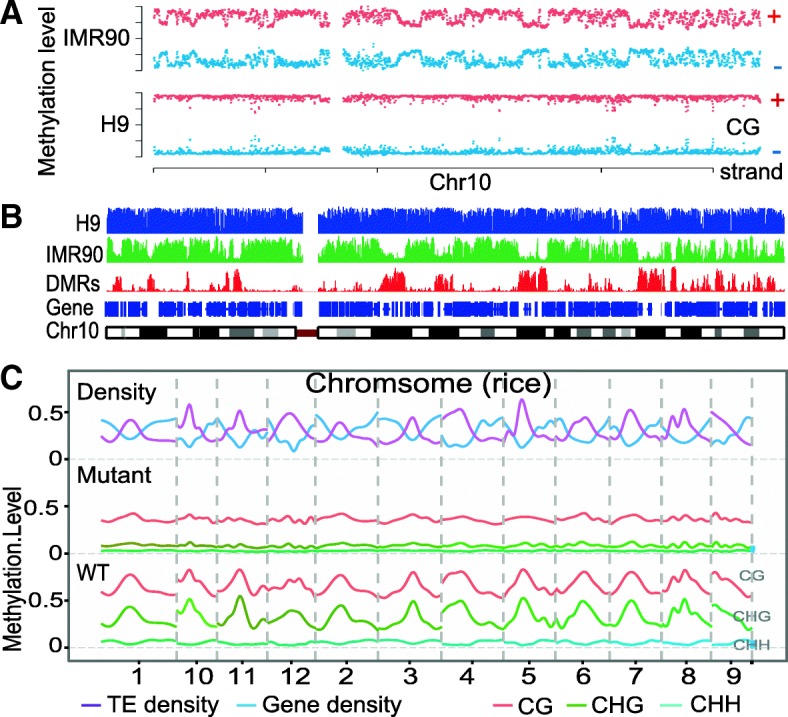
Visualization of the methylation levels in chromosome scale. **a** The methyl-cytosine density in human chromosome 10. The dots represent the methylation levels in sliding windows of 100Kb with a step of 50Kb. The red dots refer to the methylation levels in the plus strand, and the blue dots refer to the methylation levels in the minus strand. **b** An example about the distributions of the DNA methylation levels and differentially-methylated regions (DMRs) between H9 and IMR90 cell lines in human chromosome 10. **c** The density of genes, transposon elements (TEs) and the level of DNA methylation in the whole rice genome. Panel A is the results generated from Batmeth2. Panel B is the visualization results from UCSC browser, with the BED files from Batmeth2

BatMeth2 provides tools to visualize the methylation data. To illustrate the visualization features of BatMeth2, we downloaded (1) 117 Gbases of single-end reads from the human H9 cell line, (2) 105.2 Gbases of single-end reads from the human IMR90 cell line and (3) 12.6 Gbases of paired-end reads from wild-type rice. First, BatMeth2 can visualize cytosine methylation density at the chromosome level. The dots in Fig. 4a represent a sliding window of 100 kb with a step of 50 kb. To allow viewing of the ML at individual CpG or non-CpG sites in a genome browser, we also provide files in bed and bigWig formats (Fig. 4b). By comparing with the density of genes and TEs, we observed that the ML was correlated with the TE density and was anticorrelated with the gene density (Fig. 4c). This tendency has been previously observed in rice [29].

**Fig. 5:**
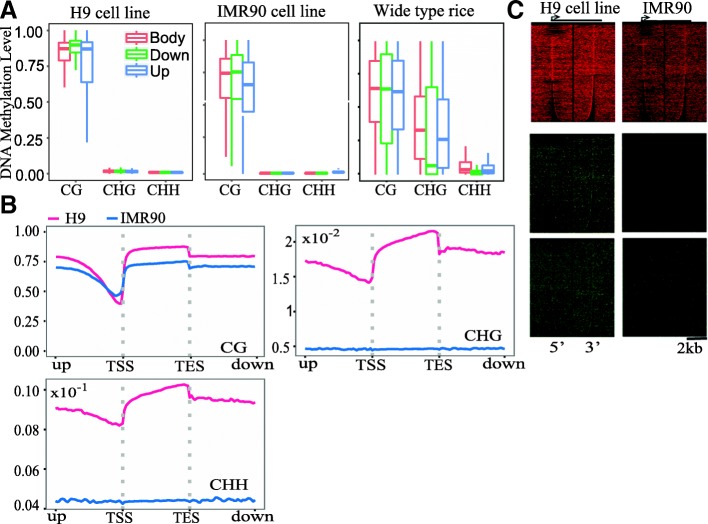
Visualization of DNA methylation under different contexts. **a** The DNA methylation levels in 2Kb regions upstream of genes, gene bodies, 2Kb downstream of gene bodies. **b** The aggregation profile of DNA methylation levels across genes. **c** The heat map of all genes in 2Kb regions upstream of genes, gene bodies, 2Kb downstream of gene bodies

Second, BatMeth2 can visualize the MLs of genes. More precisely, BatMeth2 can visualize the MLs 2 kb upstream of the gene, at the transcription start site (TSS), in the gene body, at the transcription end site (TES) and 2 kb downstream of the gene body. Comparing the upstream, body and downstream regions, Fig. 5a shows that the DNA ML of the gene body is higher than that in the promoter region. Comparing all five regions, there is obviously a valley in the TSS region (Fig. 5b). BatMeth2 can also calculate the ML profiles around introns, exons, intergenic regions and TEs (Additional file 1: Figure S4). Additionally, BatMeth2 can provide a heat map of multiple genes by gene region for convenient comparison of the overall gene MLs of different samples (Fig. 5c).

Third, BatMeth2 can visualize the distribution of DNA methylation. Additional file 1: Figure S5A shows the DNA methylation distributions in the H9 and IMR90 cell lines. In the figure, the DNA ML is partitioned into five categories: methylated (M: > 80%), intermediate between partially methylated and methylated (Mh: 60–80%), partially methylated (H: 40–60%), intermediate between nonmethylated and partially methylated (hU: 20–40%), and nonmethylated (U: < 20%). As shown in Additional file 1: Figure S5A, the ML was higher in the H9 cell line in the M category than in the IMR90 cell line, especially in the CpG context. In the CH sequence context, CpG methylation is the predominant form, but a significant fraction of methylated cytosines are found at CpA sites, while the ML is less than 40%, particularly in the H9 cell line (Additional file 1: Figure S5B).

Fourth, BatMeth2 can analyze the correlation between gene expression level and gene promoter DNA ML. We illustrated this feature using the H9 and IMR90 cell lines. The expression levels of the genes in H9 or IMR90 were divided into different categories. As shown in Additional file 1: Figure S5C, the highly expressed genes exhibited lower MLs in their promoter regions. Furthermore, we divided the MLs of the gene promoters into five categories. The result in Additional file 1: Figure S5D shows that genes with promoters having higher ML values exhibited lower expression levels. The negative correlation between the expression of mammalian genes and promoter DNA methylation is known [1]. This analysis further indicates the accuracy of BatMeth2.

### Finding differentially methylated cytosines and regions (DMCs/DMRs)

The identification of differentially methylated cytosines (DMCs) and differentially methylated regions (DMRs) is one of the major goals in methylation data analysis. Although researchers are occasionally interested in correlating single cytosine sites to a phenotype [30], DMRs are very important features [31].

Early BS-Seq studies profiled cells without collecting replicates. For such datasets, we used Fisher’s exact test to discern differentially methylated cytosines (DMCs). For BS-Seq datasets with replicates, the most natural statistical model to call DMCs is beta-binomial distribution [31]. We know that a number of software programs can perform differential DNA methylation data analysis, such as methylKit [32] (a differential analysis program that requires biological replicates) and Methy-Pipe [33] (a differential analysis program without biological duplication). However, no comprehensive package including both mapping and differential methylation analysis is available. Thus, we developed a package that integrates mapping with differential analysis. To facilitate the identification of DMRs from bisulfite data without replicates, we integrated Fisher’s exact test to perform a hypothesis test. When a sample has two or more replicates, we use the beta-binomial distribution to perform differential methylation analysis. We also provide bed or bigWig files for the list of DMRs. The DMRs can be visualized in a genome browser (Fig. 4b) with the generated bed or bigWig files.

**Fig. 6:**
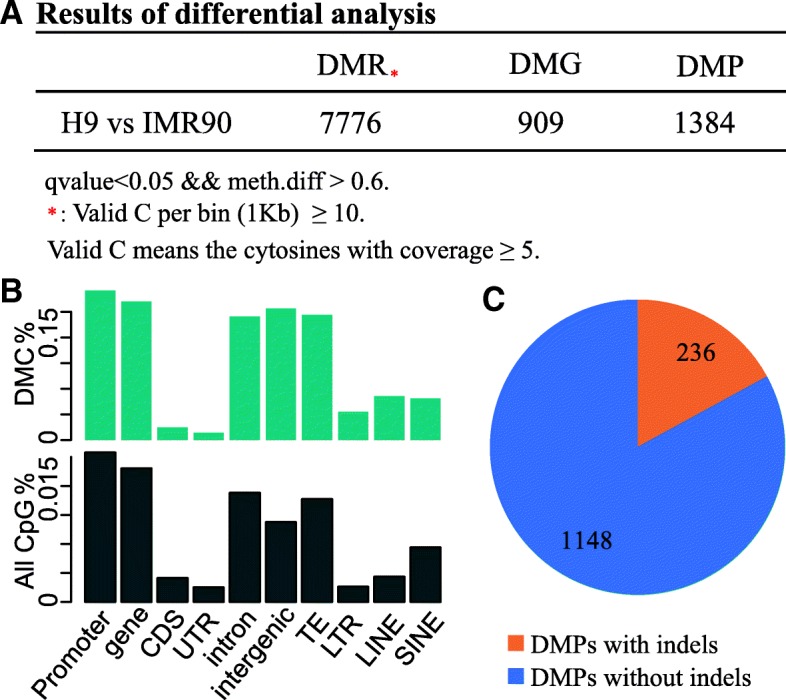
Differential methylation analysis. **a** Analysis results of differentially-methylated regions (DMRs), differentially-methylated genes (DMGs), and differentially-methylated promoters (DMPs) between H9 and IMR90 cell lines. **b** Annotation of differentially-methylated Cytosines (DMC) against different genomic properties and repeat elements. **c** DMPs contain H9 or IMR90 specific-indels (orange) occupy a substantial proportion in the all DMPs (DNA Methylation differential Promoters)

As an illustration, Fig. 6a shows the numbers of DMCs and regions in the IMR90 cell line and in the H9 cell line, as detected by BatMeth2 (*p* value< 0.05, meth.diff > = 0.6). BatMeth2 can visualize whether CpGs and DMCs are enriched in some regions, such as gene, CDS, intron, intergenic, UTR, TE, LTR, LINE and SINE regions. Figure 6b visualizes the proportions of DMCs in different genomic regions. Apart from the intergenic regions, we did not observe DMC enrichment in any regions.

### A substantial proportion of differentially methylated promoters (DMPs) contain indels

We know that indels and DNA methylation play an important role in tissue development [4] and diseases [5]. Here, we examine the relationship between differentially methylated promoters (DMPs) and indels. We performed this study using the BS-Seq reads in IMR90 and H9 cell lines. We first aligned the BS-Seq reads using BatMeth2; then, indels were called using BisSNP [34] and GATK [35] tools. Subsequently, we defined the indels that occur in only H9 or IMR90 as cell-line-specific indels.

Then, we detected 1384 DMPs between H9 and IMR90 by BatMeth2 (*p* value< 0.05, meth.diff > = 0.6). A total of 236 (17%) among all the DMPs above contain indels, as shown in Fig. 6c. In short, a substantial proportion of the DMPs contain indels. Therefore, accurate alignment of BS-Seq reads near these indels is very important for research and exploration of DNA methylation.

## Conclusion and discussion

DNA methylation plays an important role in the development of tissues and diseases. However, the complexity of DNA methylation analysis has hindered further research into the mechanism of DNA methylation in some diseases. Here, we discussed some difficulties and issues in bisulfite sequence alignment. First, incomplete bisulfite conversion when reannealing during the bisulfite conversion will lead to incorrect alignments. Moreover, sequencing errors, C-to-T converted reads and converted reference genomes further complicate the alignment of bisulfite sequences. These are the specific problems associated with aligning BS-Seq reads, in contrast to aligning normal genomic reads.

In this study, we designed and implemented BatMeth2, an integrated, accurate, efficient, and user-friendly whole-genome bisulfite sequencing data analysis pipeline. BatMeth2 improves the accuracy of DNA methylation calling, particularly for regions close to the indels. We also present a DNA methylation visualization program and differential analysis program. We believe that the superior performance of BatMeth2 should be able to facilitate an increased understanding of the mechanisms of DNA methylation in development and disease.

## Availability and requirements

**Project name:** BatMeth2.

**Project home page:** https://github.com/GuoliangLi-HZAU/BatMeth2

**Operating systems:** Linux.

**Programming Languages:** C++, Python, R.

**Other requirements:** GCC, SAMtools.

**License:** General Public License GPL 3.0.

**Any restrictions to use by non-academics:** License required.

## Additional file

Additional file 1: **Figure S1**. Outline of the mapping algorithm details. **Figure S2.** The overlap of the correct methylation callings from IMR90 cell line based on 450K BeadChip data for all compared software. **Figure S3.** BatMeth2 align BS reads allowing for variable-length indels. **Figure S3.** (A) Indel length distribution detected by BatMeth2. (B) The overlap of 450K with BatMeth2, BatMeth2 no indel detect mode and Bismark-bowtie 1(bismarkBT1). (C) More correct methylation loci in result A (mclA) covered by Indel distribution. We define the methylation sites called by BatMeth2 as Result A while the methylation sites called by BatMeth2-noIndel and Bismark as Results B. Let mclA be the methylation sites appear in Result A but not Result B. **Figure S4.** The DNA methylation level distribution across exon, intron, intergenic and TEs, etc. **Figure S5.** Methylation level under different conditions. (PDF 2044 kb)

## Abbreviation

BS-Seq: Sodium bisulfite conversion of DNA followed by sequencing
