# Supplementary material for: An integrated package for bisulfite DNA methylation data analysis with Indel-sensitive mapping
Source: BMC Bioinformatics. 2019 Jan 22;20:47. doi: 10.1186/s12859-018-2593-4 (PMC6343306; doi:10.1186/s12859-018-2593-4)
Supplement: Supplementary file 1 — Figure S1. Outline of the mapping algorithm details. Figure S2. The overlap of the correct methylation callings from IMR90 cell line based on 450K BeadChip data for all compared software. Figure S3. BatMeth2 align BS reads allowing for variable-length indels. Figure S3. (A) Indel length distribution detected by BatMeth2. (B) The overlap of 450K with BatMeth2, BatMeth2 no indel detect mode and Bismark-bowtie 1(bismarkBT1). (C) More correct methylation loci in result A (mclA) covered by Indel distribution. We define the methylation sites called by BatMeth2 as Result A while the methylation sites called by BatMeth2-noIndel and Bismark as Results B. Let mclA be the methylation sites appear in Result A but not Result B. Figure S4. The DNA methylation level distribution across exon, intron, intergenic and TEs, etc. Figure S5. Methylation level under different conditions. (PDF 2044 kb) [file 12859_2018_2593_MOESM1_ESM.pdf]

Figure S1. Outline of the mapping algorithm details.

Algorithm BatMeth2 main Algorithms.

Input: $BatMeth2$(G,R); G: Genome; R: input sequence;

Output: Alignment hits.

```
H_0 = BatMis(G_c2t|G_g2a, R, 0);
Aligns = H_0;
if Aligns > 1 then
    report;
end if
H_1 = BatMis(G_c2t|G_g2a, R, 1) ;
Aligns = Aligns + H_1;
if H_0 > 0 || Aligns > 1 then
    report;
else
    Im_0 = Search_Indels(mis_0, indels_1, |R|);
    Aligns = Aligns + Im_0;
    if Aligns > 1 then
        report;
    end if
end if
H_2 = BatMis(G_c2t|G_g2a, R, 2);
Aligns = Aligns + H_2;
if H_1 + Im_0 > 0 || Aligns > 1 then
    report;
end if
Im_1 = Search_Indels(mis_1, indels_1, |R|);
if Aligns > 0 then
    Aligns = Aligns + Im_1;
    report;
end if
Im_2 = Search_Indels(mis_2, indels_1, |R|);
if Aligns > 0 then
    Aligns = Aligns + Im_1;
    report;
end if
```

Figure S2. The overlap of the correct methylation callings from IMR90 cell line based on 450K BeadChip data for all compared software.

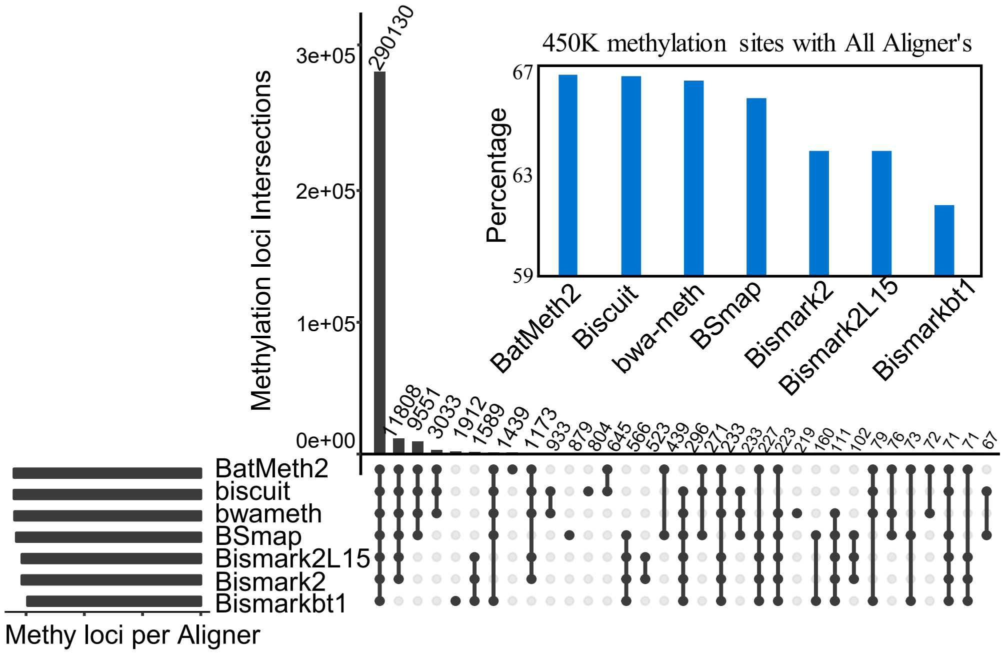

Figure S3. BatMeth2 align BS reads allowing for variable-length indels

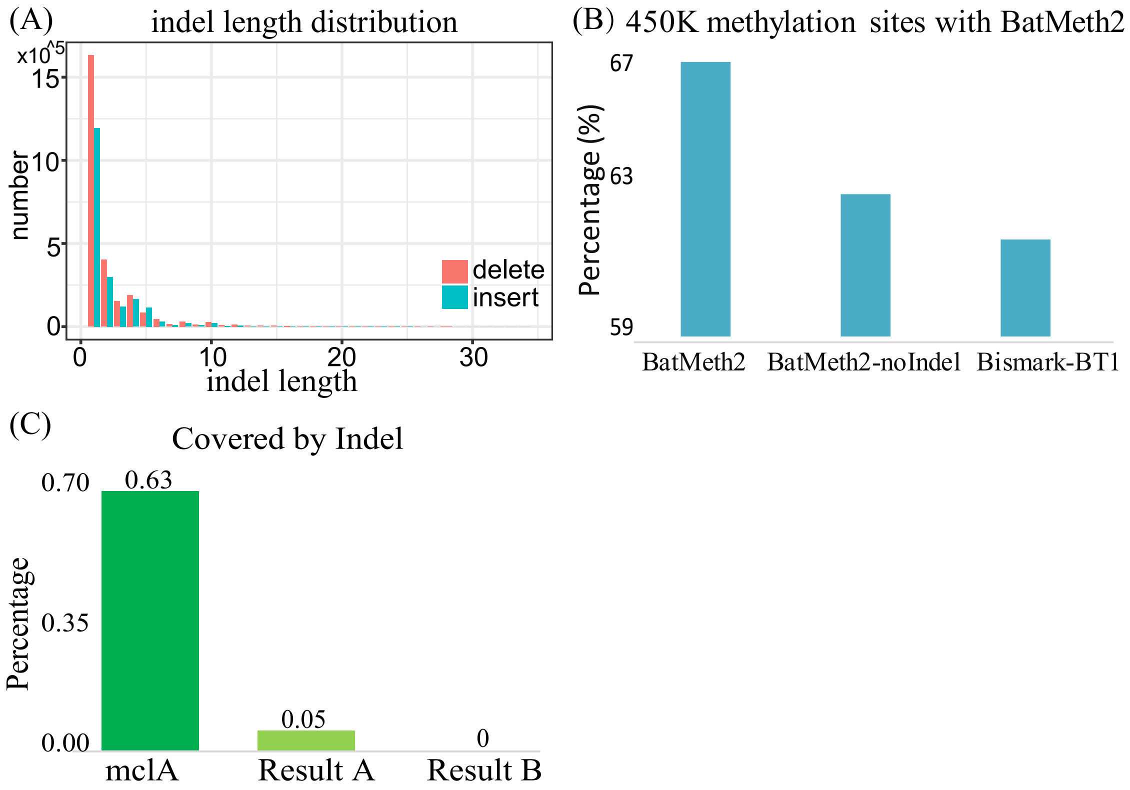

Figure S3. (A) Indel length distribution detected by BatMeth2. (B) The overlap of 450K with BatMeth2, BatMeth2 no indel detect mode and Bismark-bowtie 1(bismarkBT1). (C) More correct methylation loci in result A (mclA) covered by Indel distribution. We define the methylation sites called by BatMeth2 as Result A while the methylation sites called by BatMeth2-noIndel and Bismark as Results B. Let mclA be the methylation sites appear in Result A but not Result B.

Figure S4. The DNA methylation level distribution across exon, intron, intergenic and TEs, etc.

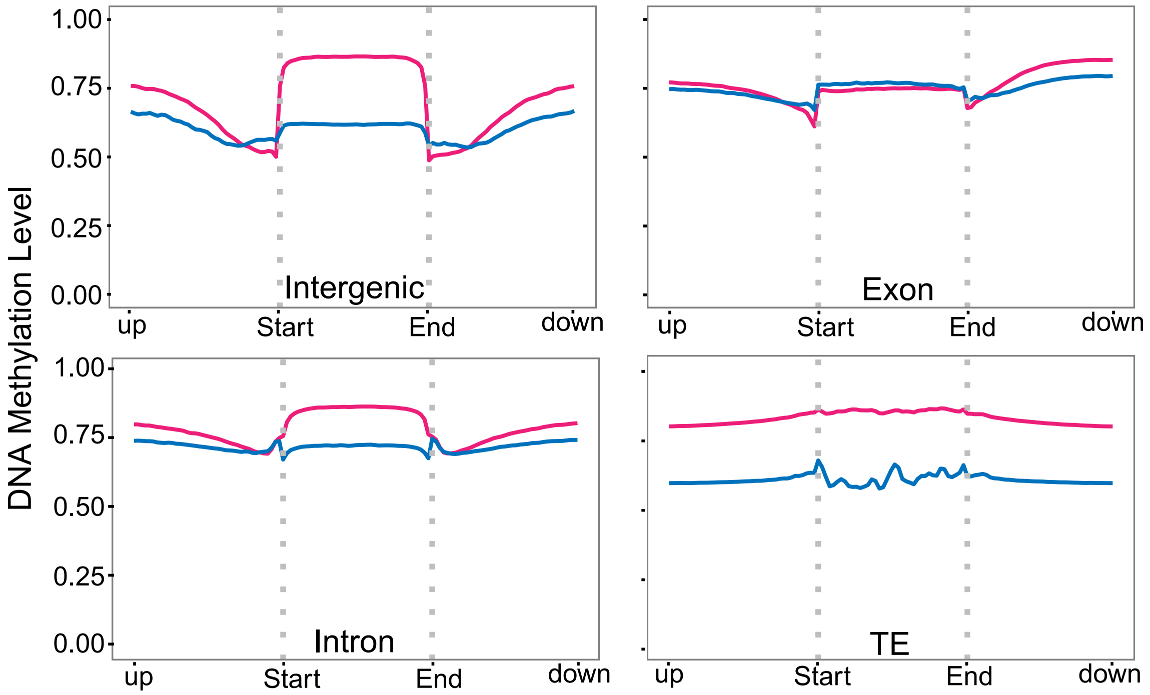

Figure S5. Methylation level under different conditions

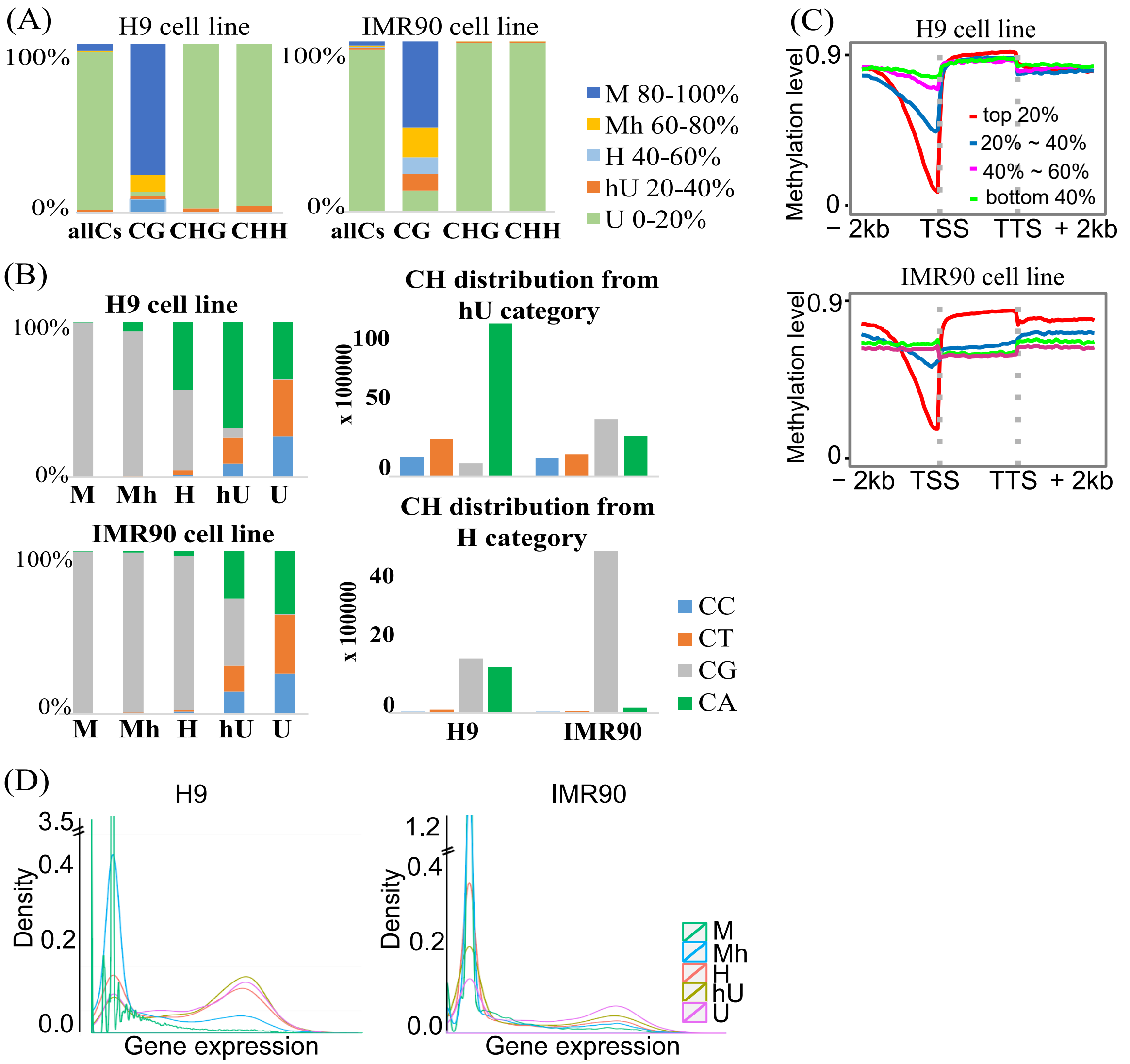

Figure S5. (A) Distribution of DNA methylation levels and the corresponding sequence context. Methylation levels were grouped into five categories: M (>80%), hM (60%<m<=80%), H 40% <m<=60%), hU (20%<m<=40%), U (<=20%). Levels of methylation were found to be highest in H9 cell line at M category than IMR90 cell line. (B) DNA methylation in various combinations of sequence contexts (CH; H = A/T/C/G) throughout the genome was examined. In the CH sequence context, CpG methylation was the predominant form, but a significant fraction of methylated cytosines were found at CpA sites while methylation level less than 40%, particularly in H9 cell line. (C) Correlation of methylation profile with expression level in H9 and IMR90 cell line. The expression levels of genes in H9 or IMR90 were divided into many categories. The higher expressed genes exhibited the lower methylation levels at the upstream from their TSS.
(D) Distribution of expression levels from genes with different promoter methylation levels in H9 and IMR90 cell lines. The methylation levels of gene promoters in H9 or IMR90 cell lines were divided into five categories. The same conclusion as C, the higher methylation levels exhibited the lower expressed level.
